# Spectral Analysis of the Slip-Length Model for Turbulence over Textured Superhydrophobic Surfaces

**DOI:** 10.1007/s10494-018-9919-1

**Published:** 2018-05-08

**Authors:** C. T. Fairhall, R. García-Mayoral

**Affiliations:** Department of Engineering, University of Cambridge, Cambridge, CB2 1PZ UK

**Keywords:** Drag reduction, Turbulent boundary layers, Superhydrophobic surfaces

## Abstract

We assess the applicability of slip-length models to represent textured superhydrophobic surfaces. From the results of direct numerical simulations, and by considering the slip length from a spectral perspective, we discriminate between the apparent boundary conditions experienced by different lengthscales in the overlying turbulent flow. In particular, we focus on the slip lengths experienced by lengthscales relevant to the near wall turbulent dynamics. Our results indicate that the apparent failure of homogeneous slip-length models is not the direct effect of the texture size becoming comparable to the size of eddies in the flow. The texture-induced signal scatters to the entire wavenumber space, affecting the perceived slip length across all lengthscales, even those much larger than the texture. We propose that the failure is caused by the intensity of the texture-induced flow, rather than its wavelength, becoming comparable to the background turbulence.

## Introduction

**Fig. 1:**
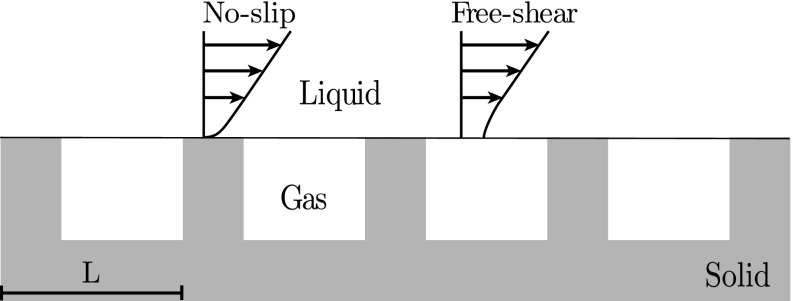
Schematic showing the mixed boundary conditions of a flow over a submerged superhydrophobic surface

Superhydrophobic surfaces combine chemical hydrophobicity with surface micro-roughness. This combination can allow pockets of gas to be entrapped between the roughness elements when the surface is submerged in water [1]. These entrapped gas pockets give superhydrophobic surfaces the ability to reduce skin-friction drag. Drag reduction using superhydrophobic surfaces has been demonstrated under experimental conditions, in laminar [2, 3] and turbulent flows [4, 5], as well as in numerical simulations [6, 7]. A reduction in drag results from the overlying flow being free to slip over the entrapped gas pockets, as shown in Fig. 1. In the limit of vanishingly-small surface texture size, i.e. where the texture size is much smaller than the turbulence structures in the overlying flow, it is reasonable to consider only the averaged effect of the surface. By considering the mean velocity at the surface the concept of a slip length is introduced through the Navier slip condition [8], defined as

1 $$ u_{s} = \ell \left| \frac{\partial u}{\partial y} \right|_{s},  $$

where *u*_*s*_ is the mean slip velocity, $\left |\partial u/\partial y\right |_{s}$ is the mean velocity gradient at the surface and *ℓ* is the mean slip length. The value of the slip length is a parameter dependent on the geometry and size of the surface texture and can be found by extrapolating the mean velocity profile from the surface to its virtual origin. Analytical solutions for the slip length have been derived for textures of streamwise and spanwise aligned ridges in the Stokes regime [9, 10] and have been obtained numerically for textures of regular arrays of posts in the same regime [11]. The slip lengths obtained in the Stokes regime have been shown to hold in turbulent flows when the slip length is small, $\ell ^{+} \lesssim 5$ [12], where the + superscript denotes scaling in wall units, based on the friction velocity, *u*_*τ*_, and kinematic viscosity, *ν*.

While drag reduction in laminar flows is solely described by the streamwise slip length, in turbulent flows the spanwise slip length, *ℓ*_*z*_, also influences the surface drag [7]. This is analogous to the drag reduction mechanism of riblets, where drag reduction was shown to be proportional to the difference between the streamwise and spanwise slip lengths [13, 14], or protrusion heights in riblet terminology. While streamwise slip increases the mean velocity, which is a drag reducing effect, spanwise slip has the effect of increasing drag, resulting in a downward shift of the logarithmic region of the mean velocity profile. This drag increase has been attributed to spanwise slip allowing quasi-streamwise vortices, which drive the near-wall turbulent cycle, to move closer to the surface [7, 14].

The classical theory of wall turbulence states that small surface modifications only influence the intercept of the logarithmic region of the mean velocity profile. In essence, the only change is the location where the outer flow experiences the wall. The Kármán constant, *κ*, and wake function remain unaffected [15]. It follows that the free-stream velocity, $U_{\delta }^{+}$ can be given by

2 $$ U_{\delta}^{+} = \left( \frac{2}{c_{f}} \right)^{1/2} = \kappa^{-1} \ \log \ \delta^{+} + B + {\Delta} U^{+},  $$

where *c*_*f*_ is the skin friction coefficient, Δ*U*^+^ is the velocity difference in the logarithmic region of the mean velocity profile compared to a smooth wall, *δ*^+^ is the flow thickness and *B* includes the smooth-wall logarithmic intercept and the wake function. When considering a boundary layer, *δ* corresponds to the boundary layer thickness. For a channel *δ* is the channel half-height, with $U_{\delta }^{+}$ the channel centreline velocity. For riblets, and for small slip lengths, it was shown that the shift Δ*U*^+^ could be given by [14],

3 $$ {\Delta} U^{+} = \mu_{0} \ (\ell_{x}^{+} - \ell_{z}^{+}),  $$

where *μ*_0_ is a coefficient found to be of order unity [13, 16, 17]. From Eq. 2, an expression for the drag reduction can be obtained,

4 $$ DR = - \frac{{\Delta} c_{f}}{c_{f_{0}}} = 1-\frac{1}{\left( 1+{\Delta} U^{+}/{U_{\delta_{0}}^{+}} \right)^{2}},  $$

where $c_{f_{0}}$ and $U_{\delta _{0}}^{+}$ are the skin friction coefficient and free-stream velocity for a reference smooth wall, respectively, and are weakly dependent on the Reynolds number.

The relationship between the spanwise slip length and Δ*U*^+^ mimics the linear relationship observed with riblets for small values of spanwise slip. However, as the spanwise slip length increases, the downward shift of the logarithmic region saturates [18]. A parametric study was carried out in [19] to map drag reduction for a range of streamwise and spanwise slip lengths. Their results can be interpreted as the spanwise effect being governed by an ‘effective’ spanwise slip length, *ℓ*_*z*,*eff*_, given by

5 $$ \ell_{z,eff}^{+} = \frac{\ell_{z}^{+}}{1 + \ell_{z}^{+} / 4}.  $$

When the spanwise slip length is small, $\ell _{z}^{+} \lesssim 1$, $\ell _{z,eff}^{+} \approx \ell _{z}^{+}$, recovering the linear relationship observed with riblets. For large values of spanwise slip, $\ell _{z,eff}^{+}$ tends to a value of 4. Due to this saturation of the spanwise effect we propose that the shift of the logarithmic region is better represented by

6 $$ {\Delta} U^{+} = \ell_{x}^{+} - \ell_{z,eff}^{+}.  $$

**Fig. 2:**
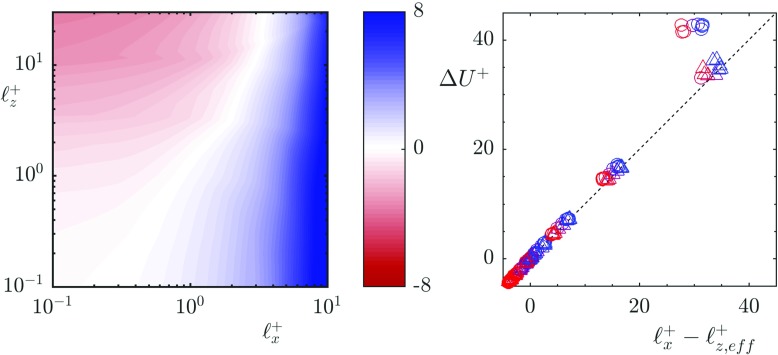
Data taken from [19] showing (left) the variation of Δ*U*^+^ with streamwise and spanwise slip lengths and (right) the linear relationship between the difference of $\ell _{x}^{+}$ and $\ell _{z,eff}^{+}$ and Δ*U*^+^. Re$_{\tau _{0}}$ = 180 (∘), Re$_{\tau _{0}}$ = 360 ($\vartriangle $), coloured from blue to red for increasing spanwise slip. The data has been rescaled by the friction velocity of each individual case

The coefficient, *μ*_0_, in Eq. 3 is here taken to be 1. Figure 2 portrays the results from [19] for Δ*U*^+^ vs. the suggested parameter $\ell _{x}^{+} - \ell _{z,eff}^{+}$, scaled by the friction velocity for each case. The results are consistent with *μ*_0_ = 1 as proposed in [13]. There is a discrepancy for large values of the streamwise slip at Re$_{\tau _{0}}$ = 180. However, at this Reynolds number relaminarisation was reported for large values of streamwise slip. Under laminar conditions, $\ell _{z}^{+}$ no longer plays an adverse effect, which would explain the discrepancy in Δ*U*^+^. Preliminary analysis by [20] suggests that saturation effect of $\ell _{z}^{+}$ is due to the impermeability imposed at the surface of these slipping simulations, which limits the shift of quasi-streamwise vortices towards the surface, an effect not present with riblets.

The theory of slip lengths, or protrusion heights assumes that the size of the surface texture is small compared to the scales in the overlying turbulent flow [21]. In this vanishingly-small limit, the overlying turbulent structures only experience the surface as the averaged effect of the texture, i.e. its slip lengths, and do not perceive individual texture elements. Slip-length boundary conditions were used in early DNS studies to predict the performance of superhydrophobic surfaces [7, 19]. The use of this model is attractive in computational simulations because additional spatial resolution to resolve the flow around the texture is not required. However, simulations of superhydrophobic surfaces with texture size of the order of scales in the overlying flow and larger exceed the vanishingly-small assumption. Consequently, the majority of recent simulations resolve the surface texture [6, 12, 22–26].

To assess the limit of applicability of the slip-length model, Seo and Mani [12] compared simulations where the texture geometry was resolved to ones where equivalent homogeneous slip lengths were instead applied. They assessed the validity of the homogeneous slip-length model for textured surfaces by measuring the instantaneous velocity and shear, spatially averaged over individual texture elements. The ratio between the two gives the instantaneous slip length over that texture element. They found that, for small textures, in their case $L^{+} < \mathcal {O}(10)$, where *L* is the texture size, as shown in Fig. 1, the equivalent slip length simulations agreed well with the textured simulations. There was a strong correlation between the velocity and shear, validating the slip-length model, and the mean velocity profiles of the homogeneous slip length simulations agreed with the textured simulations. For larger textures, however, the velocity and shear lost correlation and a large mismatch between the mean velocity profiles of the two simulations was observed.

The results of [12] suggest that the limit of the applicability of the slip length model is due to the texture size becoming of the order of scales in the near-wall turbulent cycle, specifically that of the quasi-streamwise vortices [27]. However, what is not clear is if lengthscales still much larger than the texture size, for example the near-wall streaks, still experience the averaged effect of the texture, i.e. a slip length. If lengthscales much larger than the texture size experience an apparent slip length, an extension to the slip-length model could be proposed, at least for those large scales. To investigate this possibility, in this work we consider the slip length from a spectral perspective and assess the apparent boundary condition experienced by different lengthscales in the overlying flow. The paper is organised as follows. In Section 2 our numerical method is outlined. In Section 3 we present the results from our simulations. Finally, our conclusions are summarised in Section 4.

## Numerical Method

**Fig. 3:**
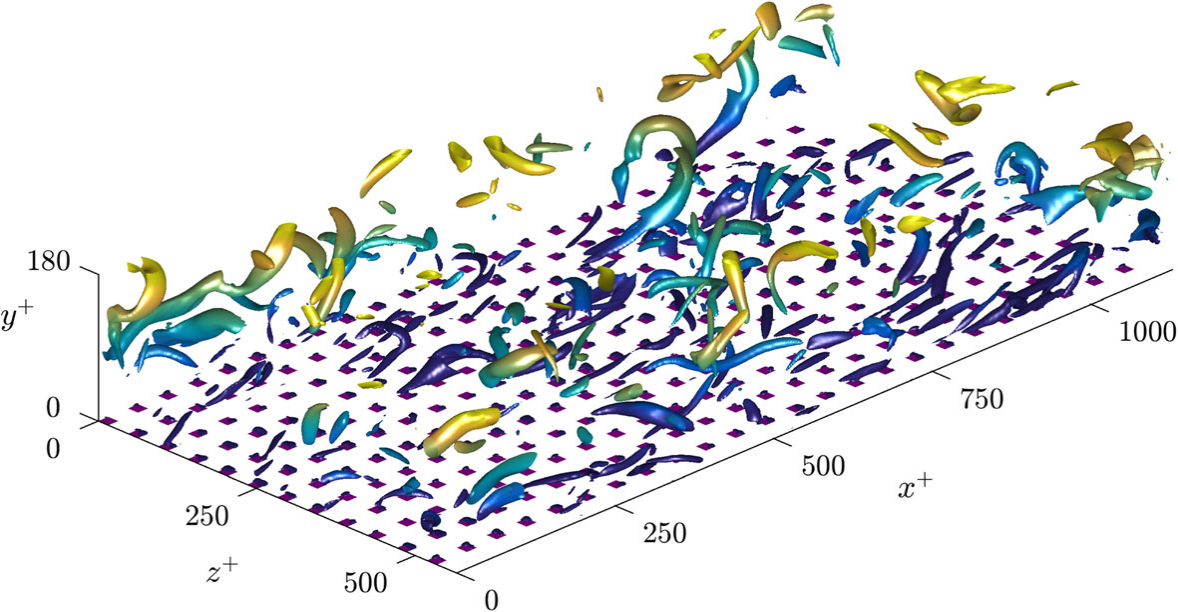
Instantaneous realisation of vortical structures, represented using the Q-criterion, showing the surface texture for the case *L*^+^ = 47

We conduct direct numerical simulations (DNS) of channels with textured superhydrophobic surfaces on both channel walls. No-slip is applied over the roughness crests, with the gas pockets modelled by a free-shear condition and considered rigid, resulting in an impermeability condition at the surface. The surface texture consists of a regular array of square posts in a collocated arrangement, shown in Fig. 3, with a solid fraction, the ratio between the area of the texture posts and total surface area, of *ϕ*_*s*_ = 1/9. This is the same texture pattern as in [12]. We study three texture sizes, *L*^+^ = 12, 24 and 47. The smallest texture size is within the range in which the slip length has previously been observed to be homogeneous. The larger texture sizes simulated are beyond the size where correlation between the velocity and shear at the surface was observed to be lost [12].

The flow within the channel is governed by the three-dimensional incompressible Navier-Stokes equations,

7 $$ \nabla \cdot \boldsymbol{u} = 0,  $$

8 $$ \frac{\partial \boldsymbol{u}}{\partial t} + \boldsymbol{u} \cdot \nabla \boldsymbol{u} = - \nabla p + \frac{1}{\text{Re}} \nabla^{2} \boldsymbol{u},  $$

where ***u*** is the velocity vector with components *u*, *v*, *w* in the streamwise, *x*, wall-normal, *y*, and spanwise, *z*, directions respectively, *p* is the kinematic pressure, and Re is the bulk Reynolds number.

The code has been adapted from [28] and is briefly summarised here. The channel is periodic in the streamwise and spanwise directions, with these directions discretised spectrally. The wall-normal direction is discretised using second-order finite differences on a staggered grid. A fractional step method [29], combined with a three-step Runge-Kutta scheme, is used to advance in time with a semi-implicit scheme used for the viscous terms and an explicit scheme for the advective terms:

9 $$ \begin{array}{llllllllll} \left[ I - {\Delta} t \frac{\beta_{k}}{\text{Re}} \text{L} \right] \boldsymbol{u}^{n}_{k} = \ & \boldsymbol{u}^{n}_{k-1} + {\Delta} t \left[ \frac{\alpha_{k}}{\text{Re}} \text{L}(\boldsymbol{u}^{n}_{k-1}) - \gamma_{k} \text{N} (\boldsymbol{u}^{n}_{k-1}) - \right.\\& \left.\zeta_{k} \text{N} (\boldsymbol{u}^{n}_{k-2}) - (\alpha_{k} + \beta_{k}) \text{G} ({p^{n}_{k}}) \right], \ k = 1,2,3, \end{array} $$

10 $$ \text{DG} ({\phi^{n}_{k}}) = \frac{1}{(\alpha_{k} + \beta_{k}) {\Delta} t} \text{D} (\boldsymbol{u}^{n}_{k}),  $$

11 $$ \boldsymbol{u}^{n}_{k + 1} = \boldsymbol{u}^{n}_{k} - (\alpha_{k} + \beta_{k}) {\Delta} t \text{G} {\phi^{n}_{k}},  $$

12 $$ p^{n}_{k + 1} = {p^{n}_{k}} +{\phi^{n}_{k}},  $$

where *ϕ* is the change in pressure, L, D, G are the discretised Laplacian, divergence and gradient operators respectively and N is the dealiased advective term operator. *α*_*k*_, *β*_*k*_, *γ*_*k*_ and *ζ*_*k*_ are the Runge-Kutta coefficients of Le and Moin [30] for the substep *k*, *I* is the identity matrix and Δ*t* is the timestep. The timestep is set by fixed CFL number CFL_a_ = 0.7 and CFL_v_ = 2.5, where CFL_a_ and CFL_v_ are the advective and viscous CFL numbers respectively, so that

13 $$ {\Delta} t = \min \left\{ \text{CFL}_{\text{a}} \left[ \frac{{\Delta} x}{\pi |u|}, \frac{{\Delta} y}{|v|}, \frac{{\Delta} z}{\pi |w|} \right], \text{Re} \ \text{CFL}_{\text{v}} \left[ \frac{{\Delta} x^{2}}{\pi^{2}}, \frac{{\Delta} y_{min}^{2}}{4}, \frac{{\Delta} z^{2}}{\pi^{2}} \right] \right\}.  $$

**Table 1:** Simulation parameters

*L* ^+^	*ϕ*_*s*_	Re_*τ*_	$N_{x_{w}}$	$N_{z_{w}}$	$N_{x_{c}}$	$N_{z_{c}}$	*N*_*y*_	*N*_*TX*,*x*_	*N*_*TX*,*z*_	$y^{+}_{int}$	$\ell _{x}^{+}$
12	1/9	180	1152	576	128	128	153	12	12	14	3.8
24	1/9	180	1152	576	128	128	153	24	24	21	6.9
47	1/9	180	576	288	128	128	153	24	24	39	10.0

*L*^+^ is the texture size in wall units, *ϕ*_*s*_ is the solid fraction, Re_*τ*_ is the friction Reynolds number, $N_{x_{w}}$ and $N_{z_{w}}$ are the number of grid points in the streamwise and spanwise directions in the refined blocks near the channel walls, $N_{x_{c}}$ and $N_{z_{c}}$ are the number of grid points in the streamwise and spanwise directions in the channel centre block, *N*_*y*_ is the number of grid points in the wall-normal direction, $N_{TX_{x}}$ and $N_{TX_{z}}$ are the number of grid points per texture element for the refined block in the streamwise and spanwise directions, $y^{+}_{int}$ is the height of the refined block above the superhydrophobic surface, and $\ell _{x}^{+}$ is the obtained mean slip length

**Fig. 4:**
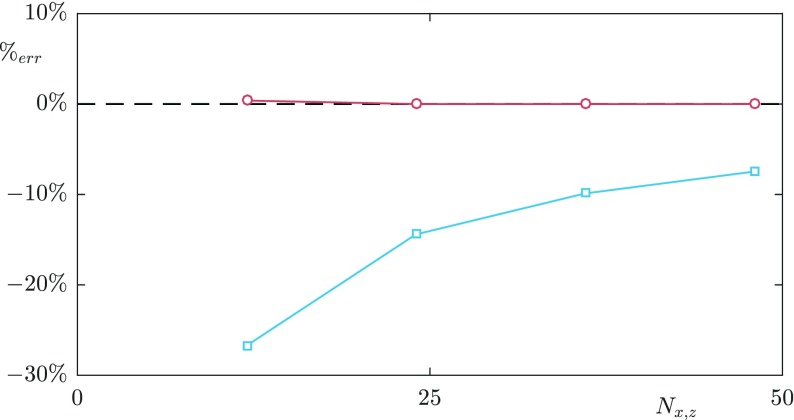
Grid resolution dependency of the mean slip length in Stokes-flow simulations. Finite difference discretisation (staggered) (); Spectral discretisation (collocated) ()

**Fig. 5:**
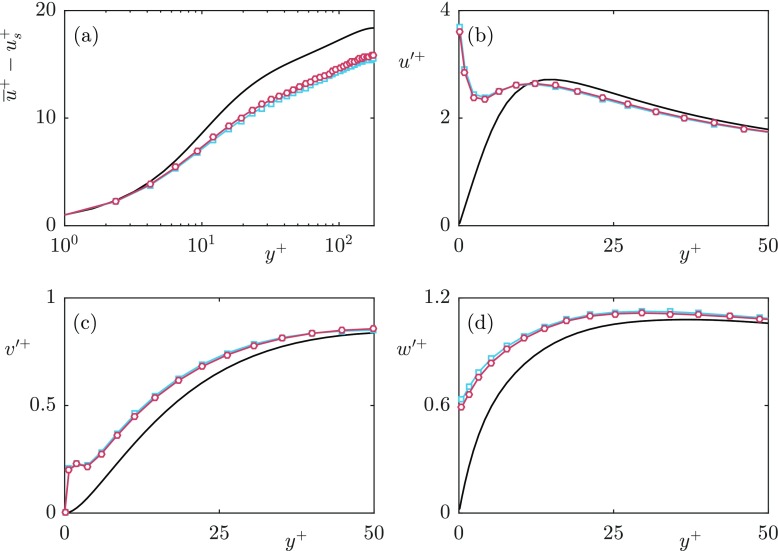
Difference between (**a**) mean velocity profiles, with slip velocity (*u*_*s*_) subtracted; **b**–**d** velocity fluctuations of the DNS grid resolution dependency test for *L*^+^ = 24. Smooth wall —; 24 points per texture (); 12 points per texture ()

To reduce the computational cost, a ‘multiblock’ grid is used which allows finer resolution near the walls. This is achieved through additional Fourier modes near the channel walls compared to the channel centre. The signal in these modes decays away from the wall, and is set to zero at the interface with the coarser, central block. It is verified *a posteriori* that the position of the interface is sufficiently distanced from the wall, so that the additional modes are not damped artificially. Further details of this multiblock grid are given in [28]. The grid resolution in the channel centre is Δ*x*^+^ ≈ 8.8, Δ*z*^+^ ≈ 4.4 with the streamwise and spanwise grid resolution near the surface dependent on the textured case, to obtain sufficient resolution for the flow induced by the texture. The grid is stretched in the wall-normal direction with resolution ${\Delta } y_{min}^{+} \approx $ 0.3 at the surfaces and ${\Delta } y_{max}^{+} \approx $ 3 in the channel centre. Full details of the simulation parameters are given in Table 1. For the *L*^+^ = 24 and 47 cases 24 grid points per texture in the streamwise and spanwise directions were used in the refined blocks near the surfaces. For the *L*^+^ = 12 case, due to the computational cost, 12 grid points per texture were used. For validation purposes, the *L*^+^ = 24 case was also run with 12 grid points per texture. From this, a grid resolution dependency on the value of the mean slip length was observed. The exact value of the mean slip length, while important for quantifying the drag reduction, is not the focus of the present work. To investigate the grid dependency of the mean slip length, we have conducted viscous Stokes-flow simulations for varying grid resolutions. A comparison for a collocated, spectral discretisation and a staggered, finite difference discretisation is shown in Fig. 4. Both simulations set-ups converge to the same value of the mean slip length with increasing grid resolution. The spectral discretisation, however, converges at a slower rate. For the spectral discretisation, using 24 grid points per texture element the difference of the obtained slip length, compared to a staggered finite difference discretisation with 64 points, is 14%, compared with a 27% difference using 12 points per texture. This is a difference consistent with the resolution dependency of the DNS results. The turbulent statistics for both texture resolutions showed good agreement, shown in Fig. 5, suggesting that the overlying turbulence is not affected by this difference in grid resolution. We therefore consider the resolutions used sufficient for the focus of the present work, although the precision of the resulting slip length is marginal.

All simulations were run at a friction Reynolds number Re_*τ*_ = 180 by applying a constant mean pressure gradient. The channel is of size 2*πδ* × *πδ* × 2*δ* in the streamwise, spanwise and wall-normal directions respectively, where *δ* is the channel half-height. Statistics were obtained by averaging over a period of 10 eddy-turnovers after statistical convergence had been reached.

While the code is spectral in the streamwise and spanwise directions, due to the discontinuous nature of the boundary conditions they are applied in physical space. The boundary conditions are implemented in the numerical method when solving the implicit part of the viscous term in Eq. 9,

14 $$ \left[ I - {\Delta} t \frac{\beta_{k}}{\text{Re}} \text{L} \right] \boldsymbol{u} =\text{RHS}.  $$

To apply the boundary conditions in physical space, following [29], the streamwise and spanwise directions and the wall-normal direction of the implicit viscous term are split and solved separately,

15 $$ \left[ I - {\Delta} t \frac{\beta_{k}}{\text{Re}} \text{L} \right] \boldsymbol{u} \approx \left[ I - {\Delta} t \frac{\beta_{k}}{\text{Re}} \text{L}_{xz} \right] \left[ I - {\Delta} t \frac{\beta_{k}}{\text{Re}} \text{L}_{y} \right] \boldsymbol{u},  $$

where L_*xz*_ includes the streamwise and spanwise components of the Laplacian, and L_*y*_ the wall-normal component. This maintains the second-order accuracy of the method [29]. The streamwise and spanwise directions are solved first, in Fourier space, and form part of the right hand side when solving in the wall-normal direction,

16 $$ \left[ I - {\Delta} t \frac{\beta_{k}}{\text{Re}} \text{L}_{y} \right] \boldsymbol{u} = \left[ I - {\Delta} t \frac{\beta_{k}}{\text{Re}} \text{L}_{xz} \right]^{-1} \text{RHS}.  $$

**Fig. 6:**
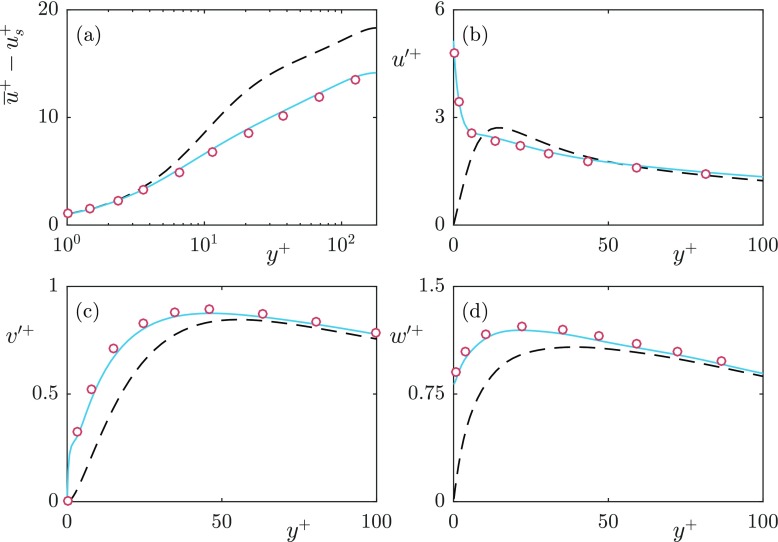
Validation of the implementation of the superhydrophobic boundary conditions against the *L*^+^ = 39 case from Seo et al. (2015). **a** mean velocity profiles, with slip velocity (*u*_*s*_) subtracted; **b**–**d** velocity fluctuations. Smooth wall (- -); Our results (); Seo et al. (2015) ()

Due to the second-order finite difference discretisation in the wall-normal direction, this forms a tridiagonal matrix equation. The implementation of the textured boundary conditions was validated against the *L*^+^ = 39 case of [25], as shown in Fig. 6.

## Results and Discussion

**Fig. 7:**
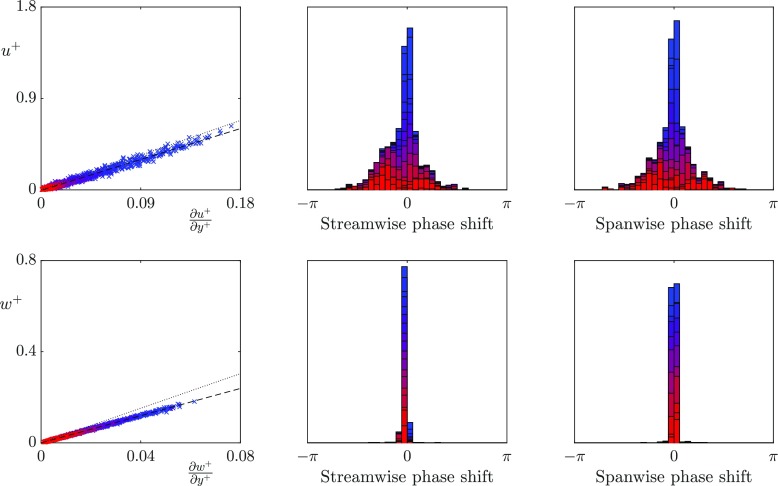
Correlation between the instantaneous surface velocity and shear for the *L*^+^ = 12 texture for wavelengths $\lambda _{z}^{+} = 94$ and $\lambda _{x}^{+} = 113-1131$ coloured from red to blue with values 1131/*α*_*x*_ where *α*_*x*_ ranges from 1–10. From left to right, velocity and shear magnitudes, streamwise phase difference between velocity and shear, spanwise phase difference between velocity and shear. Linear fit of the above data (- - -), slip length observed by the streamwise zero mode (...)

**Fig. 8:**
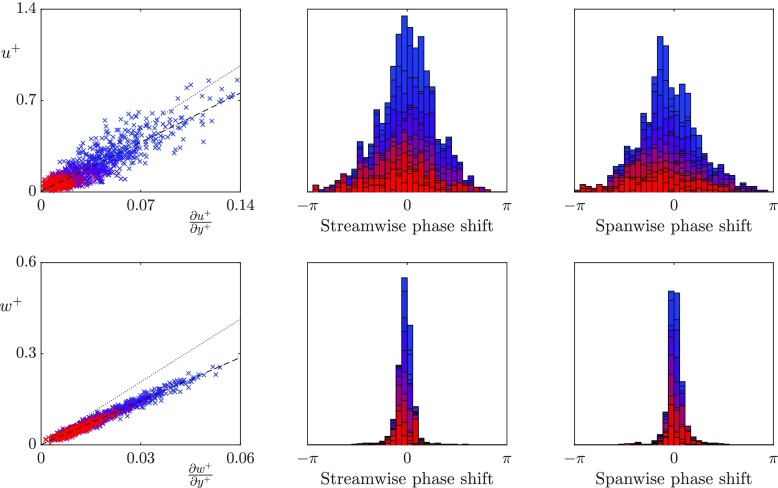
Correlation between the instantaneous surface velocity and shear for the *L*^+^ = 24 texture for wavelengths $\lambda _{z}^{+} = 94$ and $\lambda _{x}^{+} = 113-1131$ coloured from red to blue with values 1131/*α*_*x*_ where *α*_*x*_ ranges from 1–10. From left to right, velocity and shear magnitudes, streamwise phase difference between velocity and shear, spanwise phase difference between velocity and shear. Lines as in Fig. 7

**Fig. 9:**
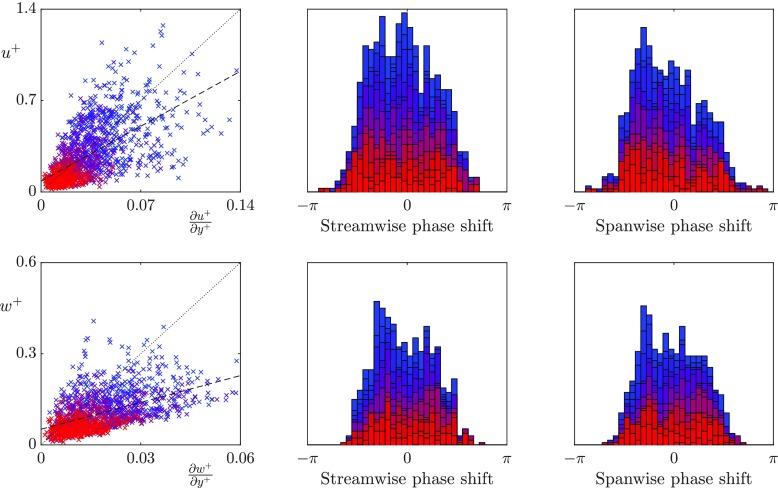
Correlation between the instantaneous surface velocity and shear for the *L*^+^ = 47 texture for wavelengths $\lambda _{z}^{+} = 94$ and $\lambda _{x}^{+} = 113-1131$ coloured from red to blue with values 1131/*α*_*x*_ where *α*_*x*_ ranges from 1–10. From left to right, velocity and shear magnitudes, streamwise phase difference between velocity and shear, spanwise phase difference between velocity and shear. Lines as in Fig. 7

We first analyse whether, for the texture sizes that have previously observed a loss of correlation, the turbulent scales that are much larger than the texture size still experience the surface as an apparent slip length boundary condition. To discriminate between the slip lengths experienced by different lengthscales in the overlying turbulent flow, we investigate the slip length from a Fourier perspective. In Fourier space, the velocity and shear contain both a magnitude and a phase, with the phase having a streamwise and a spanwise component. We analyse the instantaneous streamwise and spanwise slip lengths experienced by lengthscales larger than the texture size, and at wavelengths relevant to the near-wall turbulence dynamics. Specifically, we focus on lengthscales with a fixed spanwise wavelength, $\lambda _{z}^{+} = 94$, and streamwise wavelengths $\lambda _{x}^{+} = 113-1131$, which correspond to scales of the order of the near-wall vortices and streaks. Figures 7, 8 and 9 show the instantaneous slip lengths of these lengthscales for each texture size. For these scales to experience an apparent slip length, the instantaneous velocity and shear magnitudes should collapse to a single line, and the velocity and shear should be in phase. In the following discussion we define two slip lengths, the mean slip length and the dynamic slip length. The mean slip length, $\ell ^{+}_{x}$, is the time-average of the slip length experienced by the streamwise zero mode, i.e. it is the apparent slip length of the mean velocity profile. The dynamic slip length, $\hat {\ell }^{+}$, is the time-average slip length experienced by the fluctuations. For each case the dynamic slip length is obtained from a linear fit of the instantaneous velocity and shear fluctuations. Both slip lengths are included for reference in Figs. 7–9.

**Fig. 10:**
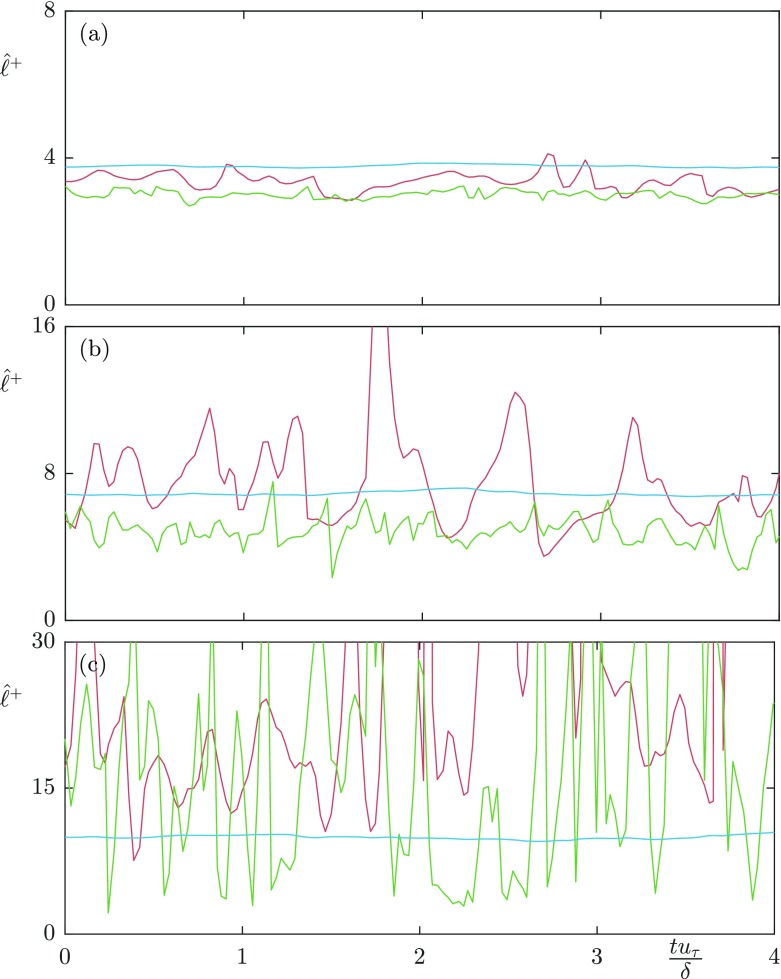
Time history of instantaneous slip lengths for **a***L*^+^ = 12 **b** *L*^+^ = 24 **c** *L*^+^ = 47. Streamwise slip length of the mean-flow (); Streamwise slip length for $\lambda _{x}^{+}$ = 1131, $\lambda _{z}^{+}$ = 94 (); Spanwise slip length for $\lambda _{x}^{+}$ = 188, $\lambda _{z}^{+}$ = 94 ()

The smallest texture size, *L*^+^ = 12, is approximately within the range of texture size where correlation between the velocity and shear has previously been observed [12]. These results agree with the previous literature, and show that the velocity and shear are strongly correlated in both the streamwise and spanwise directions. The dynamic slip lengths are $\hat {\ell }_{x}^{+}$ = 3.3 and $\hat {\ell }_{z}^{+}$ = 3.0. In comparison, the mean streamwise slip length is $\ell _{x}^{+}$ = 3.8. The fluctuations of this size therefore appear to experience a slightly smaller slip length than the mean-flow. This is consistent with the results of [12] for small textures. The lengthscales relevant to the near-wall turbulent dynamics experience no significant variation in the slip lengths.

As the texture size is increased to *L*^+^ = 24, which is of the diameter of near-wall vortices [27], there is still a strong correlation between the spanwise velocity and shear. However, the correlation becomes significantly weaker in the streamwise direction, even for lengthscales an order of magnitude larger than the texture size. These lengthscales therefore do not appear to experience the averaged effect of the texture. The time-averaged dynamic slip lengths are $\hat {\ell }_{x}^{+}$ = 5.2 and $\hat {\ell }_{z}^{+}$ = 4.8, with the mean streamwise slip length $\ell _{x}^{+}$ = 6.9. The fluctuations therefore appear to experience significantly smaller slip lengths than the mean-flow. The streamwise dynamic slip length is, however, beginning to lose physical significance due to the large fluctuations in its instantaneous value. As the texture size is further increased to *L*^+^ = 47, the correlation between velocity and shear becomes still weaker, the fitted dynamic slip lengths are for this case are $\hat {\ell }_{x}^{+}$ = 6.1 and $\hat {\ell }_{z}^{+}$ = 4.1, compared with the mean streamwise slip length $\ell _{x}^{+}$ = 10. While the dynamic slip lengths fluctuate in time, if they experienced small oscillations around a mean value, with a time-scale faster than the overlying turbulent structures perceive, then it could still be reasonable to model the surface by the time-averaged value. Time histories of the instantaneous slip length for different lengthscales are shown in Fig. 10. These show that for the largest texture, *L*^+^ = 47, the fluctuations are large compared to the mean, and their time scale is comparable to that of near-wall eddies, typically 0.1 *δ*/*u*_*τ*_ at Re$_{\tau } \simeq 180$. A time-averaged dynamic slip length is therefore not an appropriate model.

**Fig. 11:**
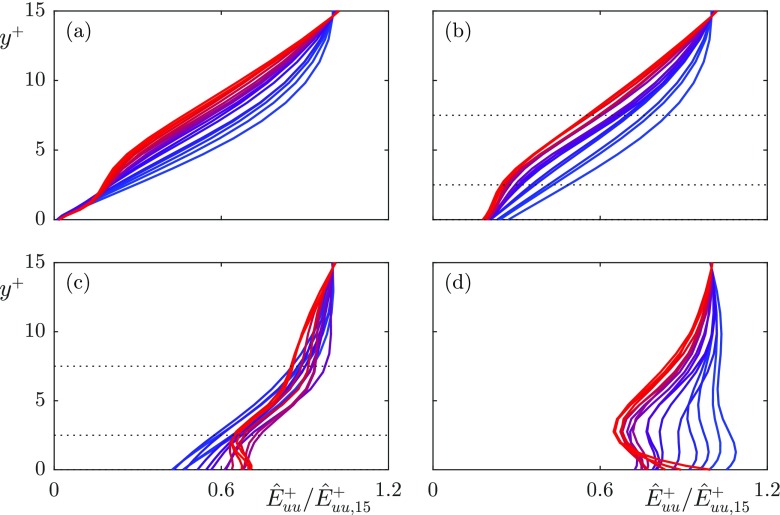
Wavelength-dependent profiles of the streamwise energy spectrum, $\hat {E}^{+}_{uu}$, normalised by their value at *y*^+^ = 15, for wavelengths $\lambda _{z}^{+} = 94$ and, from red to blue, $\lambda _{x}^{+} = 60 - 377$ with values 1131/*α*_*x*_ where *α*_*x*_ ranges from 3–19 for **a** smooth wall; **b** *L*^+^ = 12; **c** *L*^+^ = 24; **d** *L*^+^ = 47. The dotted lines indicate reference heights of *y*^+^ = 2.5 and 7.5

To investigate the source for this apparent loss of correlation we analyse the profiles in *y* of the spectral, time-averaged streamwise energy, $\hat {E}^{+}_{uu}$, for different wavelengths. Figure 11 shows these profiles for each textured case, and for a smooth wall case for reference. Since we are not concerned with the energy magnitude for each wavelength, but only with the shape of the profiles, we normalise the results with their value at a reference height of *y*^+^ = 15. The wavelengths shown correspond to $\lambda _{z}^{+} = 94$ and $\lambda _{x}^{+} = 60-377$, coloured from red to blue. As with Figs. 7–9, these are wavelengths larger than the texture wavelengths. The extrapolation of these profiles to their virtual origins gives the apparent slip length experienced by each lengthscale.

For the *L*^+^ = 12 case, the predominant effect of the surface on these energy profiles is a shift due to the slip at the surface, with the shape of the profiles essentially otherwise unmodified, compared with the smooth wall profiles. This indicates that the predominant effect of the surface, for this texture size, is the direct effect of the surface slip. For the *L*^+^ = 24 and 47 cases, however, there is a clear secondary effect near the surface, an additional energy that decays with height above the surface. This additional energy is consistent with the energy produced by the coherent flow induced directly by the texture [22, 25, 26]. Using the triple decomposition [31], the flow variables can be decomposed into a time-averaged component, a texture-coherent component and the turbulent, background fluctuations,

17 $$ u(x,y,z,t) = \overline{u}(y) + u^{\prime}(x,y,z,t) = \overline{u}(y) + \tilde{u}(\tilde{x},y,\tilde{z}) + u^{\prime\prime}(x,y,z,t),  $$

where $\overline {u}(y)$ is the mean velocity at a given height and $u^{\prime }(x,y,z,t)$ is the total fluctuation. By considering the periodicity of the texture, $u^{\prime }$ can be further decomposed into the texture-induced coherent fluctuations, $\tilde {u}(\tilde {x},y,\tilde {z})$, where $\tilde {x}$ and $\tilde {z}$ refer to the local coordinates within the texture period, and the remaining velocity fluctuation $u^{\prime \prime }(x,y,z,t)$. The velocity $\tilde {u}(\tilde {x},y,\tilde {z})$ directly results from the presence of the texture, which is periodic over texture elements. The triple decomposition has previously been used to assess the strength of the texture-induced flow over superhydrophobic surfaces [22, 25, 26].

**Fig. 12:**
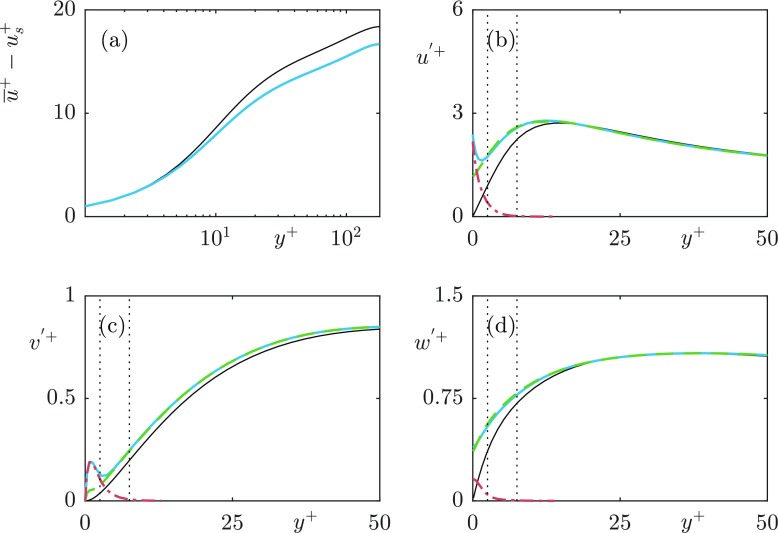
**a** Mean velocity profile with the slip velocity subtracted (**b**–**d**) rms’s of the turbulent velocity fluctuations for the case *L*^+^ = 12. Full velocity fluctuation (); turbulent fluctuation obtained from the triple decomposition (); texture-induced coherent fluctuation obtained from the triple decomposition (). The dotted lines indicate reference heights of *y*^+^ = 2.5 and 7.5

**Fig. 13:**
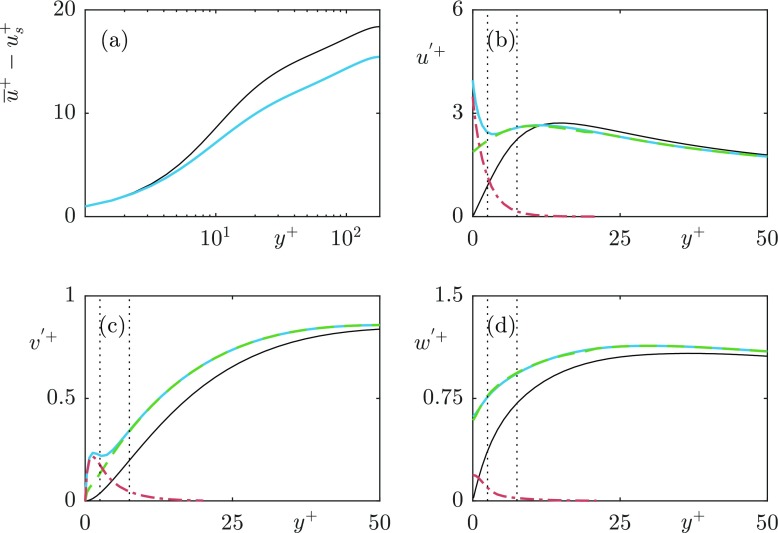
**a** Mean velocity profile with the slip velocity subtracted (**b**–**d**) rms’s of the turbulent velocity fluctuations for the case *L*^+^ = 24. Full velocity fluctuation (); turbulent fluctuation obtained from the triple decomposition (); texture-induced coherent fluctuation obtained from the triple decomposition (). The dotted lines indicate reference heights of *y*^+^ = 2.5 and 7.5

**Fig. 14:**
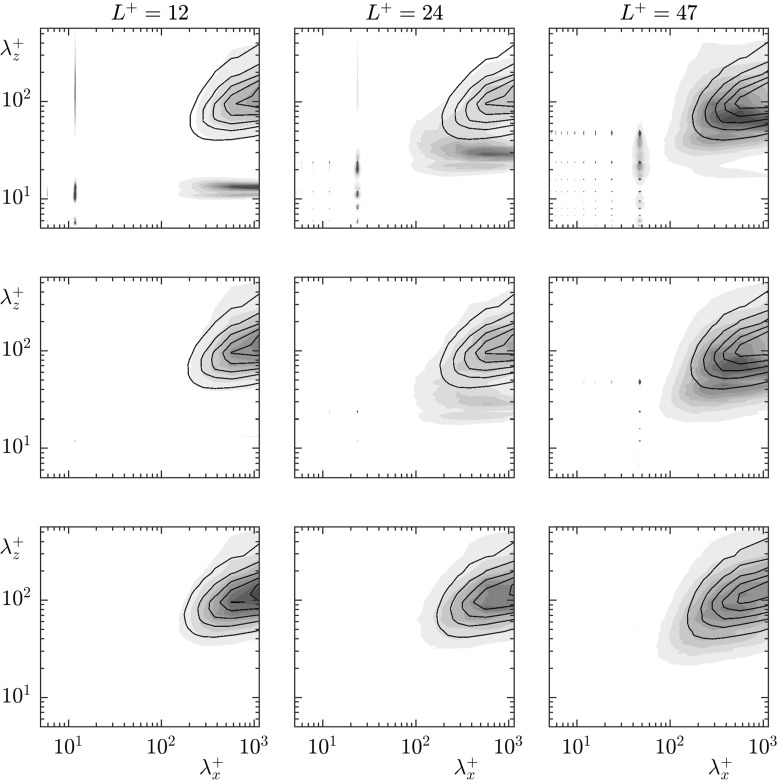
Pre-multiplied energy spectra of the streamwise velocity for the cases *L*^+^ = 12–47 at heights *y*^+^ = 0, 2.5, 7.5. Textured case (Filled contours), smooth wall case shifted by the mean slip length (lined contour)

**Fig. 15:**
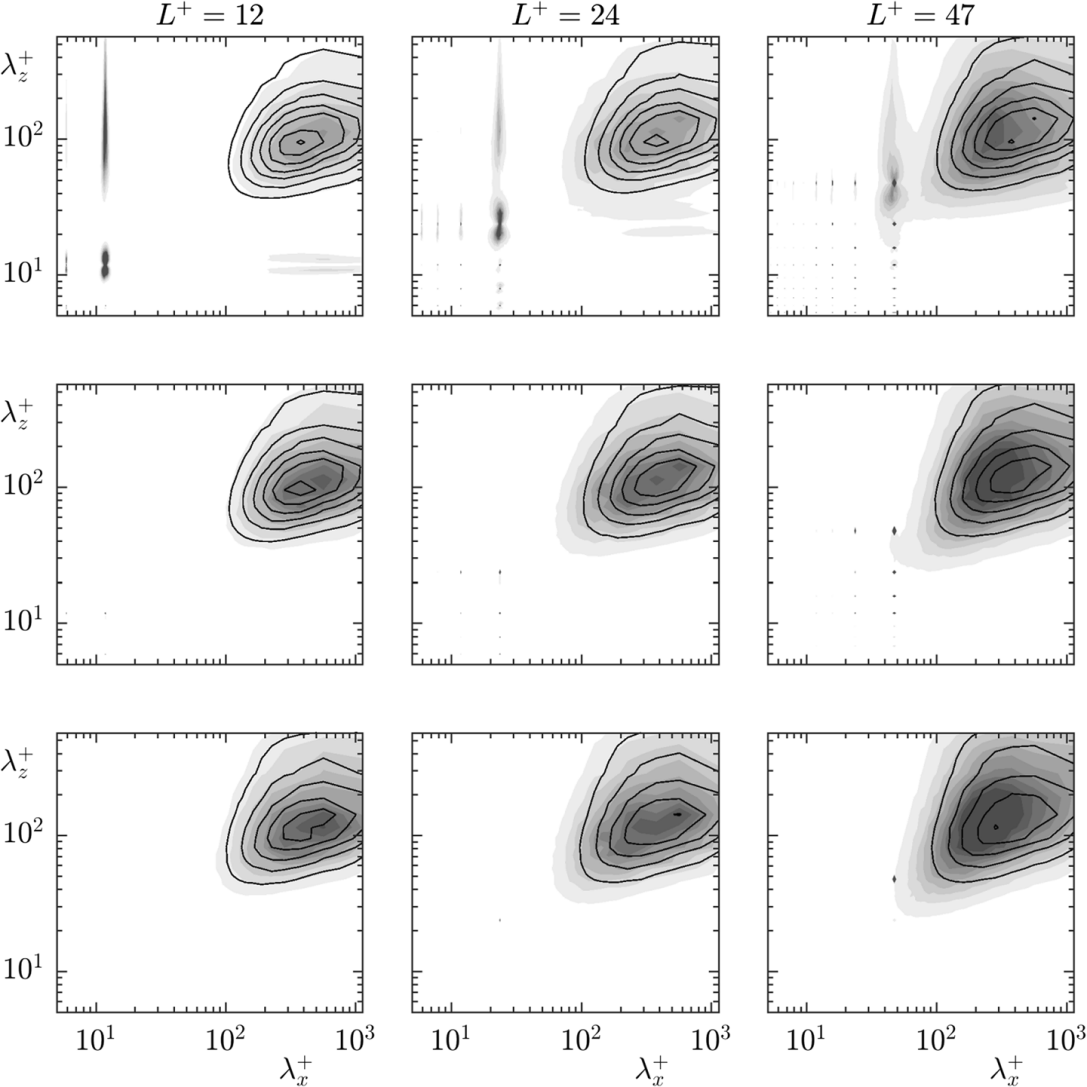
Pre-multiplied energy spectra of the spanwise velocity for the cases *L*^+^ = 12–47 at heights *y*^+^ = 0, 2.5, 7.5. Textured case (Filled contours), smooth wall case shifted by the mean slip length (lined contour)

The rms profiles of the texture-induced coherent contribution, $\tilde {u}$, for the cases *L*^+^ = 12 and 24 are shown in Figs. 12 and 13, respectively, together with $u^{\prime }$ and $u^{\prime \prime }$. The coherent contribution shows the magnitude and decay rate of the texture-induced flow, but not how the texture-induced flow is distributed in lengthscales. For this, we analyse the pre-multiplied energy spectra of the velocities. The streamwise and spanwise pre-multiplied energy spectra at heights *y*^+^ = 0, 2.5 and 7.5 for each case are shown in Figs. 14 and 15 respectively. The equivalent pre-multiplied energy spectra for a smooth wall, shifted by the mean streamwise slip length, are overlayed for comparison. Our energy spectra show energy at lengthscales consistent with smooth wall-like turbulence, and energy at lengthscales of the texture size, directly induced by the presence of the texture. In addition, however, the interaction of the texture-induced flow with the overlying turbulent flow results in texture-induced energy propagating to the lengthscales of the overlying turbulence. It is worth noting that the triple decomposition cannot remove this texture-induced flow from $u^{\prime \prime }$, as it is only able to remove lengthscales of the size of the texture [32]. From the energy spectra, it is clear that the texture-induced flow propagates across the full range of turbulent lengthscales. For texture sizes beyond *L*^+^ = 24, this interaction directly affects the turbulent lengthscales.

The magnitude of scattered energy that individual lengthscales experience depends on the texture-length scale, but more importantly on the magnitude of the texture-induced energy. For example, with the *L*^+^ = 24 case, the spanwise velocity and shear showed correlation, but the streamwise velocity and shear did not. The rms profiles and energy spectra of the velocity fluctuations show a significantly stronger energy of the texture-induced flow for the streamwise velocity compared to the spanwise velocity. The energy spectra show that the texture-induced flow of the spanwise velocity has essentially decayed by *y*^+^ = 2.5, however there is still texture-induced energy in the streamwise velocity at this height, which decays by a height *y*^+^ = 7.5. The weaker spanwise texture-induced flow scatters to the turbulent lengthscales to a lesser extent, explaining why the spanwise slip length was still correlated for this case, while the streamwise slip length was not. The upper limit of the applicability of slip-length models is, therefore, set by the magnitude of the coherent energy resulting from the texture, rather than a direct result of the texture size becoming too large.

From a Fourier perspective, the texture-induced flow can scatter to the full wavenumber space through the boundary conditions. The surface texture consists of alternating regions of no-slip and free-shear, described by wavelengths of the texture size and smaller. The boundary condition is, in Fourier space, a convolution between these texture modes and all the velocity modes. Through this convolution, the texture-induced flow can scramble to all the background turbulent modes. This scattering of the texture-induced flow to the full wavenumber space, evidenced in the pre-multiplied energy spectra, in Figs. 14 and 15, and in the energy profiles in Fig. 11, results in the measured slip length being contaminated by the texture-induced flow.

## Conclusions

We have analysed the applicability of slip-length models to represent textured superhydrophobic surfaces. Slip-length models have been used to assess the drag reduction performance of superhydrophobic surfaces when the size of the texture is within the vanishingly-small limit. In agreement with Seo and Mani (2016) [12] we show that, for texture sizes $L^{+} < \mathcal {O}(10)$, the velocity and shear are strongly correlated for the energetically relevant scales in the flow, making the slip-length model valid.

As the texture size increases, beyond the vanishingly-small limit, the velocity and shear lose correlation [12]. In this work we have assessed the slip length from a spectral perspective, to analyse whether lengthscales much larger than the texture size still experienced an apparent slip-length. We observe that when correlation is lost, this occurs across all lengthscales in the overlying flow, even those much larger than the texture size. We propose that the reason for the loss of correlation is due to the interaction of the texture-induced flow with the overlying turbulent flow which scatters the texture-induced energy into the entire flow field, contaminating the perceived slip length. We propose, therefore, that the loss of correlation between velocity and shear at the interface is caused by the magnitude of the energy of the texture-induced flow, rather than directly by the texture size exceeding the vanishingly-small limit.
